# Logistic Multidimensional Data Analysis for Ordinal Response Variables Using a Cumulative Link Function

**DOI:** 10.1017/psy.2025.10

**Published:** 2025-03-27

**Authors:** Mark de Rooij, Ligaya Breemer, Dion Woestenburg, Frank Busing

**Affiliations:** Methodology and Statistics Department, Leiden University, Leiden, The Netherlands

**Keywords:** biplots, maximum likelihood, multdimensional unfolding, MM algorithm, principal component analysis

## Abstract

We present a multidimensional data analysis framework for the analysis of ordinal response variables. Underlying the ordinal variables, we assume a continuous latent variable, leading to cumulative logit models. The framework includes unsupervised methods, when no predictor variables are available, and supervised methods, when predictor variables are available. We distinguish between dominance variables and proximity variables, where dominance variables are analyzed using inner product models, whereas the proximity variables are analyzed using distance models. An expectation–majorization–minimization algorithm is derived for estimation of the parameters of the models. We illustrate our methodology with three empirical data sets highlighting the advantages of the proposed framework. A simulation study is conducted to evaluate the performance of the algorithm.

## 1 Introduction

In many fields of study, ordered categorical variables, also called ordinal variables, are collected. In medicine, for example, patients can be classified as, say, severely, moderately, or mildly ill (Anderson & Philips, 1981). In the social and behavioral sciences, commonly Likert scales are used that have response categories such as “strongly disagree” (SD), “disagree” (D), “neutral” (N), “agree” (A), and “strongly agree” (SA). There is an ordering between these categories, but differences between these categories are unknown. It is standard practice to give numerical codes to the categories, such as 1, 2, 3, 4, 5, and subsequently perform a standard numerical analysis. In the context of regression modeling, Liddell & Kruschke (2018) argue that the analysis of ordinal response variables through linear models can lead to distorted effect sizes, inflated Type-I errors, and inversions of differences between groups.

**Figure 1:**
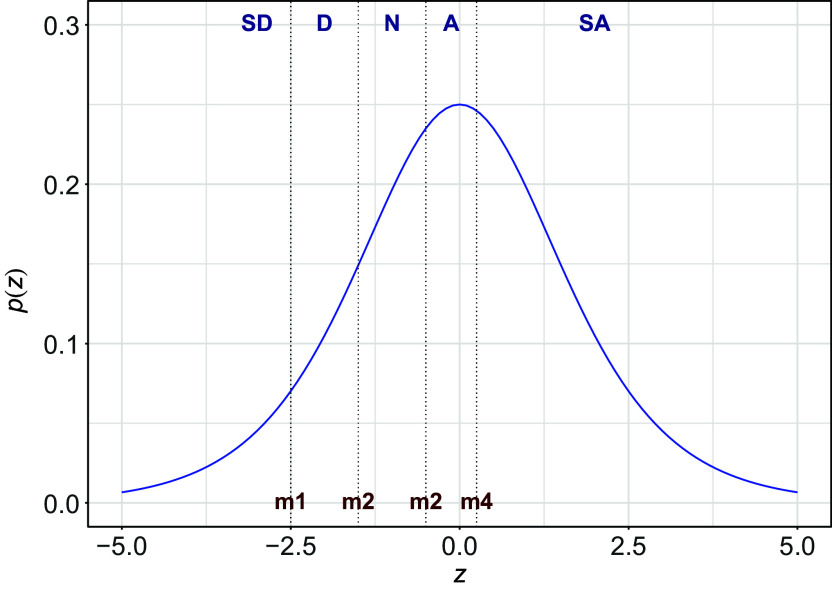
Probability density function for a continuous latent variable *z* with thresholds (indicated by the vertical lines) giving rise to an observed ordered categorical variable with categories, strongly disagree (SD), disagree (D), neutral (N), agree (A), and strongly agree (SA).

Underlying many ordinal variables, a continuous variable can be assumed. This is a *latent variable*, as we only observe the ordinal scores not the numerical ones. In Figure 1, we show the density of such a latent numerical variable. Instead of the numerical values, we observe categories such as SD, D, N, A, and SA. The continuous underlying variable is partitioned through a set of cut-points or *thresholds* into a set of categories. In Figure 1, the thresholds are shown as vertical dashed lines. All responses falling between two thresholds invoke the same response category. More formally, let *z* be the continuous latent variable. Define a set of thresholds 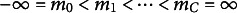 such that an observed ordinal response *y* satisfies 

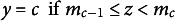

for 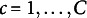 .

In regression modeling of an ordinal response variable, the following model for the latent variable is assumed (Anderson & Philips, 1981) 

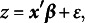

where 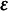 is an independent and identically distributed error term with cumulative density function *F*. This regression model for the latent response variable implies 

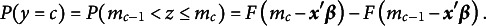

It follows that 

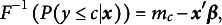

where 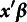 is the *structural part* of the model.

In regression modeling, the *de facto* default choice for the analysis of categorical response variables are logistic models (Agresti, 2002). For binary response variables, standard binary logistic regression models have been developed and these have been extended for ordinal variables and nominal variables (see Agresti, 2002, Chapter 7). Logistic models have the advantage that detailed interpretation in terms of changes in log-odds is possible. Such an interpretation is not available for, for example, probit models that use the cumulative density of the normal distribution. Otherwise, the fit of logit and probit models is usually very similar (Agresti, 2002, p.125). In logistic regression models for ordinal variables, we use the cumulative function of the logistic distribution, such that *F* equals 

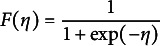

and the corresponding regression model is known as the *proportional odds model*, or, more generally, the cumulative logistic regression model (Agresti, 2002; Anderson & Philips, 1981; McCullagh, 1980; Walker & Duncan, 1967).

In many investigations, *multiple response variables* are collected. Researchers often analyze the response variables separately, but because the response variables are correlated this might not be an optimal strategy. Multidimensional data analysis refers to a set of data analysis techniques representing the multivariate data in a low dimensional, often Euclidean, space. The *R* response variables are analyzed together and the results are represented in an *S*-dimensional space, where 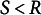 . In the low dimensional representation, the associations (i.e., correlations) between response variables are modeled.

It is important to distinguish between two types of response processes (Coombs, 1964, Chapter 1 and 26; Polak, 2011). In a unipolar or cumulative scale or map, responses are monotonically related to the position of the person on the map. The response variables are so-called *dominance variables*. Mathematical test items constitute a typical example of dominance items where subjects with a higher mathematical ability have a higher probability of solving the problem correctly. On the other hand, in a bipolar scale or map, the responses are characterized by the proximity between the variable and the respondent: The responses are single-peaked functions of the position of a variable and the position of a person. The variables are so-called *proximity variables*. For dominance variables the subjects are partitioned into homogeneous groups, that is, all subjects with a fixed response constitute a homogeneous group. For proximity items, reasons to answer totally disagree might differ between the respondents. Respondents who disagree therefore do not necessarily constitute a homogeneous group.

In classical multivariate analysis, principal component analysis (PCA, Hotelling, 1936; Jolliffe, 2002; Pearson, 1901) is the standard multidimensional data analysis tool for the analysis of dominance variables whereas multidimensional unfolding (MDU, Busing, 2010; Heiser, 1981) is the standard tool for the analysis of proximity variables.

In PCA, a data set is summarized by reducing the dimensionality using a set of *principal components*, that are linear combinations of the original variables. The principal components explain as much of the original variability as possible. PCA solutions can be graphically represented through so-called biplots (Gabriel, 1971; Gower & Hand, 1996; Gower et al., 2011), where the row objects (observations, participants, individuals) are represented as points in a Euclidean space and the columns (variables, items) as vectors or variable axes. The projection of the points onto the axes is informative.

Another multidimensional data analysis approach is MDU. MDU is targeted toward proximity variables and uses a distance representation. MDU solutions can also be graphically represented by biplots, where both the row objects and the variables are represented by points in a Euclidean space. The distances between the two sets of points are informative.

When besides the response variables also predictor variables are available with information about the row objects, we can constrain the PCA or MDU to incorporate this information. The principal scores or the ideal points are restricted to be (linear) functions of the predictor variables. When we constrain PCA in such a manner, the resulting model is known as reduced rank regression (RRR; Izenman, 1975; Tso, 1981) or redundancy analysis (Van den Wollenberg, 1977). RRR models can be represented graphically, by so-called triplots (Ter Braak & Looman, 1994). When we constrain MDU in such manner, we obtain restricted MDU (RMDU; Busing et al., 2010). These RMDU models can be graphically represented by triplots.

PCA, MDU and its constrained versions, RRR and RMDU, are usually estimated by least squares methods. For categorical response variables, however, linear models estimated with least squares methods are not optimal and might lead to distorted effect sizes, type-I errors, and inversion of effects (Liddell & Kruschke, 2018). Logistic models estimated using maximum likelihood offer an alternative.

For binary data, several authors (De Leeuw, 2006; Landgraf & Lee, 2020; Schein et al., 2003) proposed PCA using the binomial negative log-likelihood as loss function. Collins et al. (2001) proposed a generalization of PCA to the exponential family to deal with, for example, binary data or integer-valued data such as count data. As far as we know, only Vicente-Villardón & Sánchez (2014) investigated exponential family generalizations of PCA for ordinal response variables including a biplot visualization.

For constrained PCA of binary variables, that is, RRR or redundancy analysis, a logistic model was proposed by De Rooij (2024). Yee & Hastie (2003) generalized RRR to response variables from the exponential family, similar to generalized linear models (McCullagh & Nelder, 1989). As far as we know, there are no exponential family generalizations of these reduced rank models for ordinal response variables.

For MDU, several attempts can be found in the literature to exponential family MDU models. Andrich (1988), Takane (1998), and DeSarbo & Hoffman (1986) defined MDU models for binary variables using squared distances. Andrich (1988) proposed a unidimensional model that does not allow for predictor variables. Takane (1998) and DeSarbo & Hoffman (1986) describe generalizations to multiple dimensions that can include predictors. De Rooij et al. (2025) defined a model on the basis of (unsquared) distances for binary data, both with and without predictor variables. As far as we know, there are no exponential family generalizations of MDU for ordinal response variables.

In conclusion, there have been several attempts to define exponential family models for PCA and MDU and their constrained versions that include predictor variables. These attempts mainly focus on binary response variables, but some include also other types of variables (Collins et al., 2001; Yee & Hastie, 2003) like count variables. However, no exponential family generalizations exist of PCA or MDU for ordinal response variables, neither with or without predictor variables.

The goal of this article is to fill this gap and propose multidimensional models for ordinal response variables in the exponential family. We will develop models for multivariate ordinal dominance variables (i.e., PCA and RRR) and proximity variables (i.e., MDU and RMDU). The user has to choose between these two approaches. Models without predictor variables, i.e., PCA and MDU, and with predictor variables, i.e., RRR and RMDU, will be presented. Along the algebraic formulation, we will also develop biplot methodology for visualization of the models. One unified algorithm for maximum likelihood estimation of model parameters will be developed and tested, where at the lowest level updates differ between the four approaches. To illustrate the multidimensional models and the difference between the dominance and proximity perspective, we will apply the models on several data sets. In the first application, we have cognitive data and use the dominance approach. The second application highlights the difference between the dominance and proximity approaches using behavioral response variables. The third application shows in detail the proximity approach where the response variables concern attitudes concerning the environment. The second and third application use data from the International Social Survey Programme (ISSP Research Group, 2022). Biplots for all three applications will be discussed in detail.

The outline of this manuscript is as follows. In Section 2, we propose a family of geometric, multidimensional, models for multivariate ordinal data. We distinguish between dominance and proximity response variables and between models with and without predictor variables. Properties of the model are derived. We briefly discuss model selection and discuss in detail the visualization of the models using biplots. In Section 3, we present a unified algorithm for maximum likelihood estimation of model parameters. In Section 4, we show the three applications. We test our algorithm using simulated data in Section 5. We end this article with some discussion and conclusions.

## 2 Cumulative logistic multidimensional models

We consider a set of ordinal variables with observed values 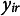 ( 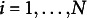 , 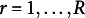 ) where variable *r* has 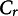 categories, coded as 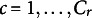 . Underlying each ordered categorical response variable 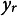 we assume a continuous latent variable 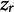 . We model these latent variables as 

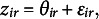

where 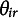 , the structural part of the model, is geometrically defined in *S* dimensions. When using PCA we define 

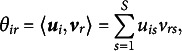

with 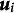 the principal scores and 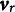 the loadings, whereas 

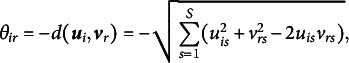

with 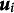 the point for participant *i* and 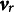 the location for variable *r* when MDU is used. Note that the 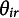 are negative by definition when distances are used but can be positive or negative with the inner products. We denote the two models by cumulative logistic PCA (CLPCA) and cumulative logistic MDU (CLMDU). The 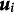 and 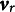 can be collected in matrices, that is, 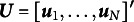 and 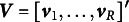 .

When predictor variables are available for the participants, the coordinates of the principal scores or ideal points can be restricted to be a linear, additive function of these predictor variables, that is, 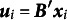 with 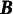 a 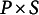 matrix. Within the PCA context, we obtain the RRR model (CLRRR). For MDU, we obtain a RMDU (CLRMDU).

We assume the 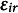 to be independent and identically distributed error terms following a cumulative logistic distribution. The probability density function of the logistic distribution equals 

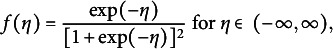

such that its logarithm is 

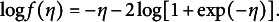

The cumulative function of this distribution equals 

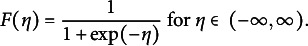

It follows that 

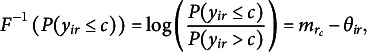

where, similar to the proportional odds regression model, the thresholds ( 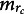 ) are *category specific*, but the structural part ( 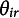 ) of the model is *variable specific*.

### 2.1 Properties of cumulative logistic models

Let us consider two subjects with locations 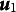 and 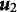 . The cumulative log-odds ratio for response variable *r* is defined as 

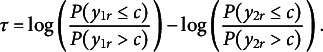

With a PCA (or RRR) parameterization, 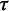 can be written as 

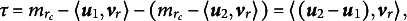

which does not depend on *c*. This shows that CLPCA (and CLRRR) make a *proportional odds assumption*, for a given change in the positions for the subjects all the cumulative log-odds for variable *r* change with the same amount. Furthermore, if we define 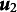 as 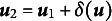 , such that 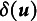 is a shift from one position to another, then we may write 

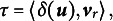

which shows that it does not matter where in the Euclidean space this shift happens, the cumulative log-odds ratio remains constant for constant 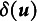 . Consider participants 1 and 2, with coordinates 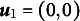 and 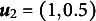 . The estimated cumulative log-odds ratio for these two participants is the same as for participants 3 and 4 with coordinates 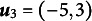 and 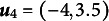 , as the difference between these pairs of coordinates is the same.

When predictor variables are used in the analysis, we constrain the coordinates to be linear combinations of those variables, that is, 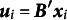 . In this case, the comparison between two persons that differ one unit in one of the predictor variables but have equal values otherwise is of interest. Say, the *p*th predictor variable increases by a unit, such that, 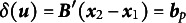 , then the cumulative log-odds increase by 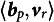 .

Below in Section 2.3, we will describe biplots for the interpretation of our multidimensional models. Cumulative logistic RRR (CLRRR) models can also be interpreted numerically, similar to the regression weights in a proportional odds model. With reduced rank coefficient matrix 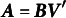 , each column of this matrix represents a change in cumulative log odds for the corresponding response for unit increase in the predictor.

For cumulative logistic MDU and restricted MDU, there is a nonlinear distance relationship. The cumulative log-odds ratio becomes 

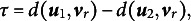

again not depending on *c*, only on the distances. This is again the proportional odds assumption, but in contrast with the PCA parameterization, however, the changes in cumulative log odds are not constant for changes in 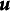 . That is, using the example with participants 1, 2, 3, and 4, described above again, the cumulative log-odds ratio for participants 1 and 2 is not equal to that of participants 3 and 4. Similarly, unit changes in one of the predictors do not lead to a constant change in cumulative log-odds (i.e., the estimated 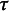 is different for two participants with predictor values 0 and 1 compared to two participants with predictor values 2 and 3, say). Furthermore, unit changes in one of the predictors (say, 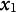 ) lead to different changes in 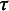 for participants with varying values on the other predictors. Although the relationship of the predictors is additive in defining the positions of the participants in the biplot, the relationship between predictors is not additive when looking at the effect on the response variables. Results can therefore not be represented numerically and we have to rely on the biplot visualizations described in detail below.

### 2.2 Model selection

Assuming conditional independence between the response variables given the representation in low-dimensional space, we will estimate the model parameters by maximizing the likelihood (see Section 3, where we derive an algorithm). Model selection entails (1) selecting a good dimensionality and (2) in case there are predictor variables, selecting the set of predictor variables that have an effect on the response variables.

For maximum likelihood methods there are several type of statistics that can be used for inference. The best known statistics are Wald tests, likelihood ratio tests, and information criteria like Akaike’s Information Criterion (AIC; Akaike, 1974). However, for our cumulative logistic multidimensional models, we need to make the following observations. For Wald statistics, we need standard errors of the parameters. Such standard errors are not a by-product of our MM-algorithm (next section). For obtaining standard errors and/or confidence intervals the non-parametric bootstrap can be used (Efron, 1979; Efron & Tibshirani, 1986). The likelihood ratio statistic compares two nested models. If the model under the null hypothesis is true and certain regularity conditions are satisfied, the likelihood ratio statistic is known to be asymptotically distributed as a chi-square variable with degrees of freedom equal to the difference in the number of parameters under the two hypotheses. For our models, there are two complications: (1) The regularity conditions are not satisfied for selecting the optimal dimensionality, see Takane et al. (2003) and Takane & Van der Heijden (2023) for a detailed discussion; (2) we generally do not belief a certain model to be true as this involves many assumptions.

Therefore, in this article we will use information criteria for model selection, that is, the AIC and BIC. For the AIC and BIC we need the number of parameters. In all our models, we have the threshold parameters, the number of which is 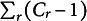 . For PCA and RRR, the number of parameters in the structural part is 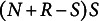 and 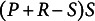 , respectively. For MDU and RMDU, the number of parameters in the structural part is 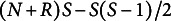 and 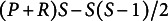 , respectively.

For the models with predictor variables, we follow the suggestion of Yu & De Rooij (2013) to use a step-wise approach to reduce the computational load. In the step-wise approach, we start with defining the matrix 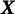 to include all predictors of interest and determine the dimensionality *S*. Second, using the just selected *S*, we search for an optimal set of predictors by iteratively leaving out columns of 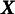 .

### 2.3 Biplots

In this section, we will discuss biplots for the visualization of the model results. These biplots are most valuable for two-dimensional solutions, but can also be used for visualization of pairs of dimensions in case of higher dimensional solutions. We first discuss biplots for CLPCA and CLMDU. Afterwards, we discuss the case when predictor variables are available for the analysis. In that case, the biplots are extended with extra information about the predictor variables and become triplots.

#### 2.3.1 CLPCA biplots

**Figure 2:**
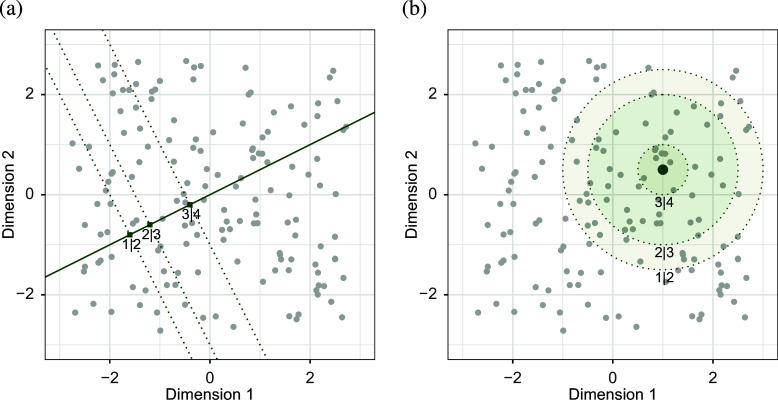
Biplot representations for CLPCA (left) and CLMDU (right) for a single response variable.*Note*: Variable markers for cumulative probabilities are added. Grey points represent observations. On the left, the green solid line represents the variable axis with markers indicating the estimated thresholds. The dotted lines indicate decision regions for the categories of the response variable. On the right, the green point represents the response variable. The circles represent decision boundaries, where outside the circle the first category of the label is preferred and inside the circle the second category of the label.

Biplots (Gabriel, 1971; Gower & Hand, 1996; Gower et al., 2011) are useful displays for the results of a PCA, especially for two-dimensional solutions. We will now discuss the geometry of the two-dimensional biplot for CLPCA. Like a usual PCA biplot, observations are shown as points, and variables are shown by axes. The coordinates of the points are given by the estimated 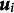 . The variable axes are straight lines though the origin with direction 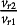 . In Figure 2a, we present a simplified biplot where the observations are shown by grey dots and there is a single variable axis (solid line). For this variable, 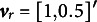 and suppose 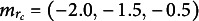 for 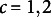 and 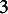 , respectively, i.e., thresholds for a four-point response scale. Estimated values for the response of an individual can be obtained by projecting the point representing this individual onto the variable axis. Subjects positioned in the lower left corner have lower expected values for the response, while subjects in the upper right corner have higher expected values, projecting higher onto the variable axis. To further increase interpretation and provide numerical values for the expected value, Gower & Hand (1996) suggest to add labeled markers to the variable axis. For CLPCA there are several possibilities.

Vicente-Villardón & Sánchez (2014) suggest to add markers based on the largest estimated a posteriori probabilities. That is, for every point on the variable axis the probability for each response class is computed. At specific locations on the variable axis there are points where two categories, say *c* and 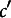 , jointly have the highest probability. These points are marked as with 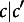 . They note that in some cases the probability of one or several categories are never higher than the probability of the other categories. When, for example, category 2 is such a “hidden” category, the marker will be “1|3”.

In the context of the proportional odds model, Anderson & Philips (1981) suggest to make predictions based on the underlying latent variable. Following this suggestion, we propose to add markers based on the underlying latent variable and the estimated thresholds. The predicted value of the latent variable is 

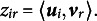

As in standard PCA, this inner product is constant ( 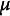 , say) for all points on a line projecting at the same location of the variable axis, that is, a line orthogonal to the variable axis. Therefore, the point of projection may be calibrated by labeling this point with the value 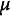 . This value also applies to the point of projection itself, which is 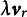 for some 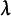 . For the point 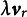 to be calibrated with the value 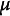 , it must satisfy 

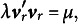

so that 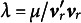 . The coordinates of the point on the variable axis that is calibrated with a value of 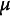 are 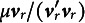 . As such, we would have the markers expressing values of the underlying latent continuous variable, but the interest lies in the observed ordinal response variable. The estimated response is 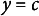 if 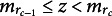 . Therefore, markers can be based on the estimated thresholds. These markers indicate the transition points between adjacent categories. The coordinates of the marker point are given by 
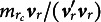
 and these can be labeled by 1|2, 2|3, and so forth. The application of these markers is illustrated in Figure 2a. An advantage of these markers over the ones based on posterior probabilities is that each threshold is represented.

Based on these markers, the two-dimensional space can be partitioned into 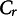 areas by drawing 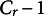 decision lines orthogonal on the variable axis and through the marker points, these are represented by the dotted lines in Figure 2a.

In our explanation above, we focused on a single response variable. The proposed cumulative logistic models, however, are methods for multiple response variables. Therefore, in the biplots a variable axis is presented for each response variable. Each variable axes is accompanied with a variable label which we position at the positive end, that is, the side with highest scores of the variable axis. These variable axes for the set of variables jointly partitioning the multidimensional space in open and closed regions that each represent a particular predicted response profile.

#### 2.3.2 CLMDU biplots

In MDU biplots, both the observations and the variables are shown by points in the two-dimensional space. The closer an observation to the variable point the higher the probability of a high response. To represent the ordinal nature of the response variable into the biplot, remember that we have 

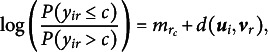

so that 

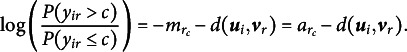

It follows that we can add circles to the biplot with center 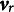 and radius 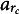 , such that for points inside this circle the probability for responding higher than *c* is larger than 0.5 while outside the circle this probability is smaller or equal to 0.5. Every variable point is therefore accompanied with 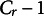 circles representing the different probabilities. We illustrate the threshold circles in Figure 2b, where again 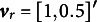 and 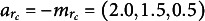 . We see again that the two-dimensional space is partitioned in several areas. These areas are now defined by the circles. Within the smallest circle around the response variable, the participants are predicted to score highest. Around the inner circle, we have regions in the form of tyres. Within such a band, participants are predicted to have the same score on the response variable. The final region is the region outside the circles. For participants whose points are located in this region we predict the lowest scores.

In some cases a radius can be negative (when 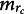 is positive), indicating that nowhere in the low-dimensional space the corresponding cumulative probability is larger than a half, and consequently the circle is not drawn.

These CLMDU models are also defined for multiple response variables. In the corresponding biplots each response variable is represented by a point and a set of circles. Circles of different response variables cross each other, creating regions that correspond to predicted response profiles.

### 2.4 Restricted models

When predictor variables are available for the observations, the principal scores or participant points ( 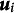 ) are defined to be linear combinations of the predictor variables, that is, 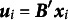 . To include the predictor variables in the biplot, we distinguish between numerical and categorical predictor variables. Numerical predictor variables are included in the two-dimensional biplot as variable axes, that are, straight lines through the origin with direction 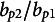 . Markers and labels indicating typical values for the predictor variables are added to these variable axes. Furthermore, also variable labels are added to the biplot and placed at the end of the variable axes corresponding to the highest scores. The positions of the participants can be obtained from the predictor variable axes by the process of *interpolation*, as outlined by Gower & Hand (1996). The interpolation process is similar to vector addition, that is, we create vectors starting in the origin and along the variable axes to the observed predictor value for each predictor variables. To obtain the position of the participant, we have to add these vectors.

Categorical predictor variables, are recoded into dummy variables, where one of the categories is chosen as a reference category. In the biplot representation, we use points instead of variable axes for such predictor variables. The position of the reference category is in the origin of the low-dimensional space, whereas the other categories are positioned at their corresponding estimates in 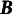 . We may consider categorical predictor variables as “jumps.” When the categorical predictor is equal to the reference category, no jump is made. When the categorical predictor variable is equal to another category, a jump from one position to another is made. These jumps are on top of the interpolation process for numerical predictor variables. When multiple categorical predictor variables are included in the analysis, multiple jumps need to be made to find the corresponding position of a participant.

Variable axes for the numerical predictor variables and points for the categorical predictor variables are added to the biplots for CLPCA or CLMDU. For interpretation, the relationship between a predictor variable and a response variable is of interest. In the CLRRR biplots, for numerical predictor variables such a relationship is given by the angle between a predictor variable axis and that of a response variable (see Section 2.1). A sharp angle indicates a strong relationship, while an obtuse angle indicates a weak relationship. Furthermore, for every point along the predictor variable axis, a projection onto the response variable axis can be made to obtain a predicted value. For a categorical predictor, the point representing a category can be projected onto the response variable axis to obtain a predicted value.

For CLRMDU biplots, the interpretation of predictor-response relationships is more involved, because these are single-peaked where with increasing values of a predictor first the response goes up and afterwards down again. Furthermore, although the effect of predictor variables is additive for obtaining the position of an observation (i.e., the point 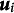 ), this additivity does not translate to the relationship toward the response variable.

## 3 Maximum likelihood estimation

Assuming a multinomial distribution of the response variables, the *observed data negative log-likelihood* is 

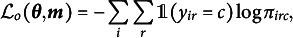

where 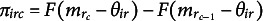 , and 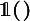 is an indicator function of its argument, 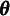 collects all the structural parameters and 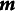 collects all the threshold parameters.

In this section, we will develop an expectation–majorization–minimization (EMM) algorithm to minimize the negative log-likelihood. This algorithm is a combination of the EM-algorithm often used for latent variable models (McLachlan & Krishnan, 2007) and the MM-algorithm (Heiser, 1995; Hunter & Lange, 2004).

We start by formulating the complete data negative log-likelihood, take the conditional expectation of this function in the E-step, and find a majorization function that can easily be minimized. The majorization function turns out to be a least squares function, so that in the inner loop of the algorithm, well known updating steps from least squares theory can be used.

### 3.1 Estimation of structural part

The complete data negative log-likelihood is defined for the latent responses, that is, 

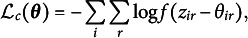

where 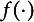 is the probability density function of the logistic distribution (see Section 2). A second-order Taylor expansion of the complete data negative log-likelihood around current values 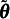 is 

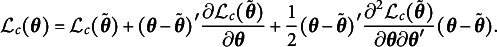



In the E-step, the expectation of the complete data negative log-likelihood is obtained, that is, 

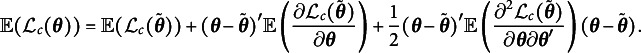

As the expectation of a sum is the sum of expectations, we can write 

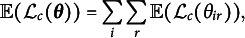

with 

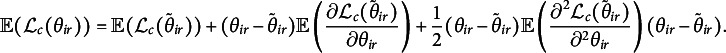



Let 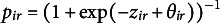 such that 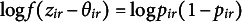 . The partial derivative is 

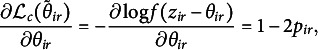

and the second order derivative is 

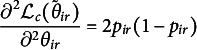

which is bounded from above because 
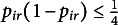
 . Therefore, 

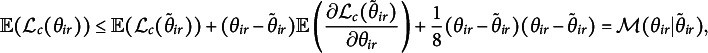

where 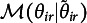 is the majorization function.

We need the expectation of 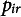 to find the expectation of this partial derivative. Following Jiao (2016), we derived the following closed form expressions (we use *p*, *y* and 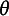 instead of 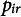 , 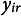 and 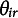 for readability): 

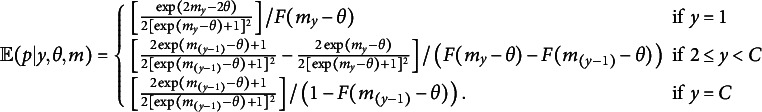

The expectation has to be evaluated at the current estimates of 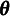 and 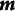 . Let us denote by 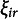 the expected value of the first derivative, that is, 

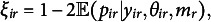

so that the majorization function to be minimized is 

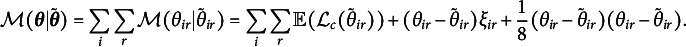

Let us simplify this majorization function. Focusing on the individual elements, the first term is a constant ( 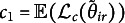 ) and therefore 

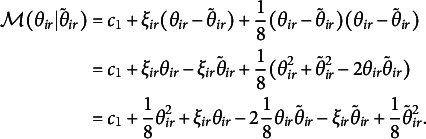

Now, let us define what we will call *working responses*

(1)

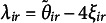

to obtain 

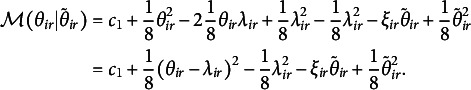

Define 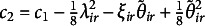 , a constant with respect to 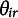 , so that we can write 

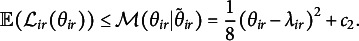

Now collecting all terms into a single function, we obtain 

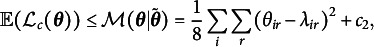

a least squares function with the working responses 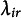 . In the following four subsections, we work out this least squares loss function for the four different definitions of the structural part ( 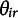 ).

#### 3.1.1 PCA parametrization of the structural part

Remember that 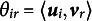 , so that the loss function equals 

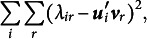

which can be written in matrix algebra terms as 

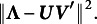

We have to find a reduced rank approximation of the matrix 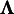 with elements 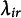 . Eckart & Young (1936) showed that this can be done using a singular value decomposition 

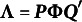

and defining the updates 
(2)

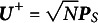



(3)

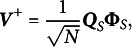

where 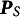 and 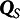 are the first *S* singular vectors, and 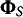 is the 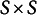 diagonal matrix with the largest singular values.

#### 3.1.2 RRR parametrization of the structural part

Compared to the PCA parametrization, we impose the constraint that 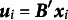 . Therefore, in each iteration, we have to minimize 

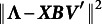

over the parameters 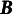 and 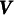 . Updates of 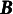 and 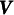 can be obtained from a generalized singular value decomposition of the matrix 

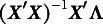

in the metrics 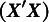 and 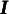 (Takane, 2013, Section 2.3.6). Let 

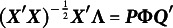

be the usual SVD. The updates are defined as 
(4)

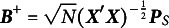



(5)

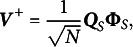

where 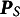 and 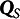 are the first *S* singular vectors, and 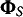 is the 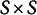 diagonal matrix with the largest singular values.

#### 3.1.3 MDU parametrization of the structural part

The loss function in every iteration is 

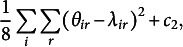

where 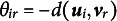 with 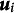 and 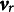 the parameters. This loss function can be rewritten as 

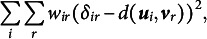

where 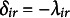 and 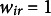 , which is the usual raw STRESS function often used in multidimensional scaling and unfolding. De Leeuw (1977) and De Leeuw & Heiser (1977) proposed the SMACOF algorithm for minimization of this STRESS function for multidimensional scaling. The SMACOF algorithm is itself an MM algorithm. Convergence properties of this algorithm are described by De Leeuw (1988). Heiser (1981, 1987) showed that MDU can be considered a special case of multidimensional scaling. Subsequently, he developed the SMACOF algorithm to deal with rectangular proximity matrices. Advances in the algorithm are described in Busing et al. (2010, Chapter 8). An elementary treatment of the algorithm for multidimensional scaling can be found in Borg & Groenen (2005) and for MDU in Chapter 14. The critical difference with the usual loss function is that the dissimilarities 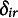 might be negative. Heiser (1991) showed a way to deal with negative dissimilarities in multidimensional scaling. The line of thought of Heisers contribution is that two majorizing functions are defined: one for the case that the dissimilarity is positive and one for the case that the dissimilarity is negative. It turns out that the new algorithm is a simple adaptation of the standard SMACOF algorithm, where only some elements of two matrices ( 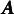 and 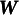 , see below) are defined differently, depending on the sign of the dissimilarity. De Rooij & Busing (2024) and De Rooij et al. (2025) adapted Heiser’s algorithm for the MDU case. Here, we will follow that approach. We will only show the updating equations, for the derivation of these equations, we refer to the above papers.

Define matrix 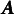 with elements 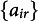 as follows 

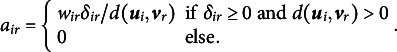



Furthermore, redefine the weight matrix 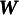 with elements 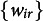 as 

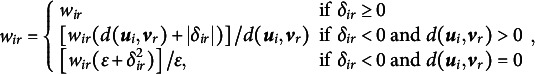

where 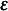 is a small constant. Note that the matrices 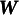 and 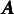 change from iteration to iteration.

Let us now define 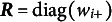 , 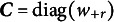 , 
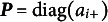
 , and 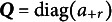 , where the + in the subscript means taking the sum over the replaced index, that is, 
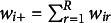
 . With these matrices the update for 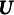 is 
(6)

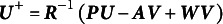

and for 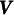 the update is 
(7)

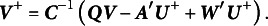

These updates are the same as in the standard least squares unfolding algorithm (see Busing, 2010, pp. 176, 183–187), where only the definitions of 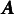 and 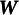 are changed.

#### 3.1.4 RMDU parametrization of the structural part

When predictor variables are available, we constrain 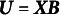 and we need to estimate 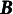 instead of 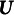 . We can use the algorithm of MDU described in the previous section and replace the updating equation for 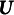 (i.e., Equation (6)) with an updating equation for 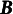 , that is, 
(8)

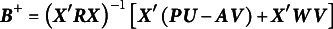

and before updating 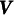 using Equation (7), we compute 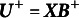 .

### 3.2 Estimation of thresholds

To update the threshold parameters for each response variable we cannot use the complete data negative log-likelihood. However, we can use the default maximum likelihood estimator as in the proportional odds regression model. For this estimator 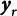 is the response variable and we use 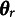 as an offset, that is, a predictor variable with regression weight fixed to 1 and without any other predictor variables. This gives maximum likelihood estimates of the intercepts or thresholds for response variable *r*. We repeat the procedure for each response variable.

### 3.3 Remarks on algorithms

The algorithms as outlined above monotonically converge to a local minimum of the negative log-likelihood function. For the models based on the inner product (PCA and RRR) this local minimum is also the global minimum. For models based on the distance representation, however, local optima occur. To deal with these local optima, starting values near the global minimum might help, such as given by, for example, correspondence analysis. As such a start does not guarantee to find the global minimum, supplementary multiple random starts are advised.

### 3.4 Algorithm schemes

To summarize, we give algorithm schemes in four algorithm boxes. Algorithm 1 shows the procedure for CLPCA, algorithm 2 for CLRRR, algorithm 3 for CLMDU, and algorithm 4 for CLRMDU.

**Figure:**
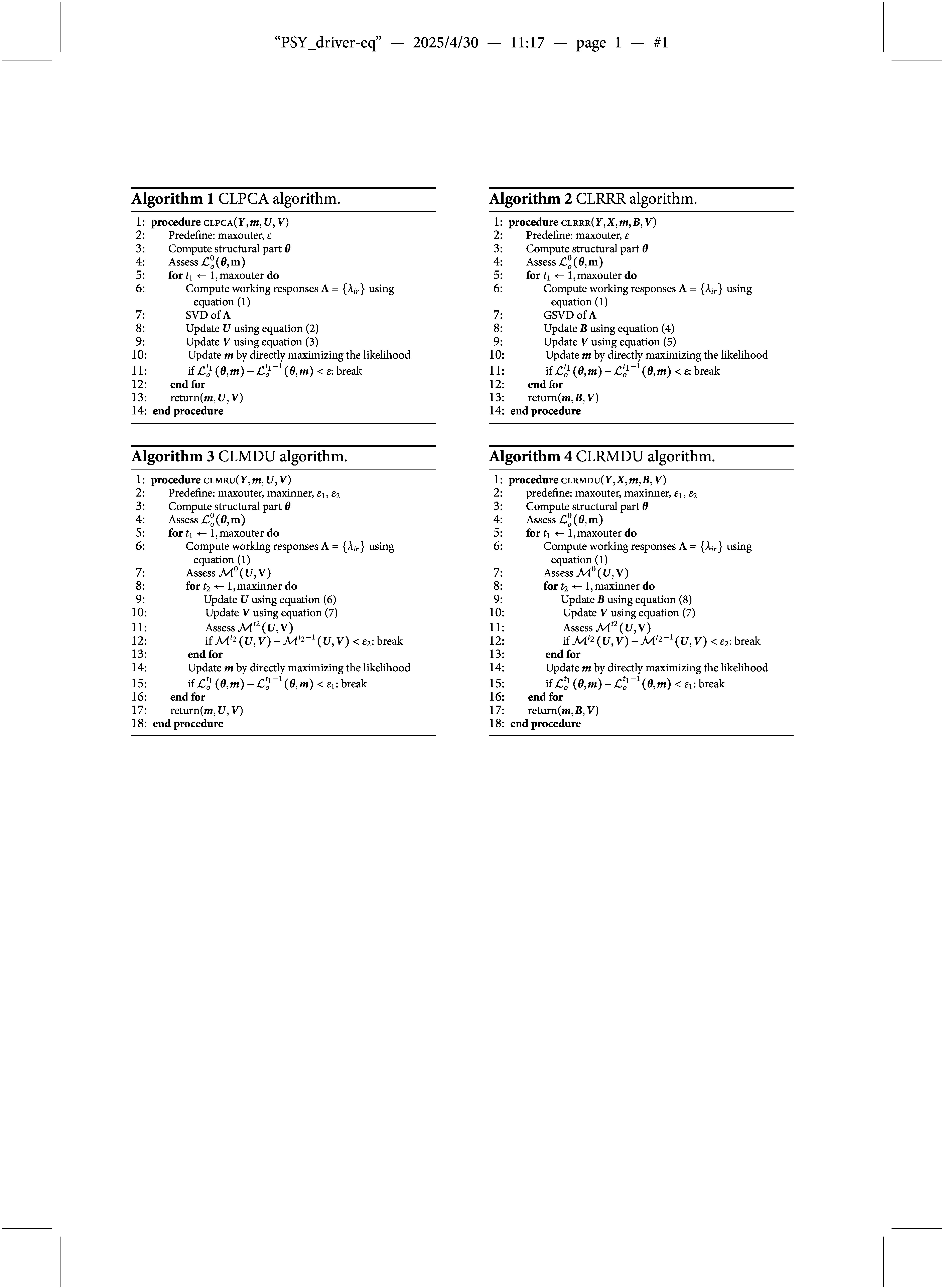

## 4 Applications

In this section, we will show three applications of our modeling framework. In the first application, we analyze part of the data described by Fabbricatore et al. (2024) about students responses to exam questions about statistics. We focus on the effects of several psychological variables like attitude and anxiety on these responses. As the response variables are cognitive items, we will analyze these data using the cumulative logistic reduced rank model. In the second application, we use data from the ISSP to investigate the relationship between environmental attitudes and pro-environmental behavior. In this analysis, we use both the dominance and proximity models to highlight the differences between the two approaches. Finally, we show a third application again using data from the ISSP. We use data from seven Likert scales to investigate differences between countries in environmental efficacy. As the response variables are attitude items, we use the cumulative logistic restricted multidimensional unfolding (CLRMDU) model for this analysis.

### 4.1 Students’ performance for statistical tests

The subset of data we use for this analysis involves 138 university students and their responses to ten questions in an exam about the application of statistics to certain topics. The ten items are described in detail in Appendix A. Each response is coded as wrong (0), partially correct (1), or correct (2), that is, as a three categories ordinal response.

Prior to the start of the courses on statistics, several psychological tests were conducted. Besides their answers on the ten items, for each student information is available on their gender (1 = female, 0 = male), age in years, and several measurements of mathematical knowledge and psychological factors, each assessed through validated psychometric instruments. The variables that we use are:

- *Mathematical knowledge* measured using the Mathematical Prerequisites for Psychometrics scale (PMP);
- *Statistical anxiety* with three scale scores referring to examination anxiety (SASa), interpretation anxiety (SASi), and fear of asking for help (SASf);
- *Attitudes toward Statistics* with four scales: affect (SATSa), cognitive competence (SATSc), value (SATSv), and difficulty (SATSd);
- *Motivated strategy for learning* with four scales referring to self-efficacy (MSLe), test anxiety (MSLt), cognitive strategies (MSLc), and self-regulation (MSLsr);
- *Academic procrastination* measured using a single scale (APS);
- *Academic motivation* also measured using a single scale (AMS);
- *Student engagement in statistics* measured through three scales: affective engagement (ENGa), behavioral engagement (ENGb), and cognitive engagement (ENGc).

Psychometric properties of these scales are all satisfactory to good, see Fabbricatore et al. (2024) for details, where also more detailed descriptions of the scales can be found. The scores on these scales serve, together with gender and age, as predictor variables. The responses to the ten statistical items serve as response variables. The research questions that are central to these data is whether the psychological factors influence the responses on the exam items and whether these effects are homogeneous for different exam items or heterogeneous.

As there is no prior information whether the 10 items comprise a unidimensional or multidimensional construct, we will start fitting models in one till three dimensions and select an optimal dimensionality. Subsequently, we will verify which of the predictor variables influence the responses. Fit statistics for the one-, two-, and three-dimensional models are shown in Appendix A, where it can be seen that the two-dimensional model has the lowest AIC, while the lowest BIC is obtained for the unidimensional model. We proceed with the two-dimensional model. Leaving out each (set of) predictor variables, we obtain the fit statistics in the lower part of Table A1, showing that Age, Statistical Anxiety, the Motivated Strategies for Learning Questionnaire, and the Academic Motivation Scale can be left out without significant loss of fit (AIC based conclusion).

**Figure 3:**
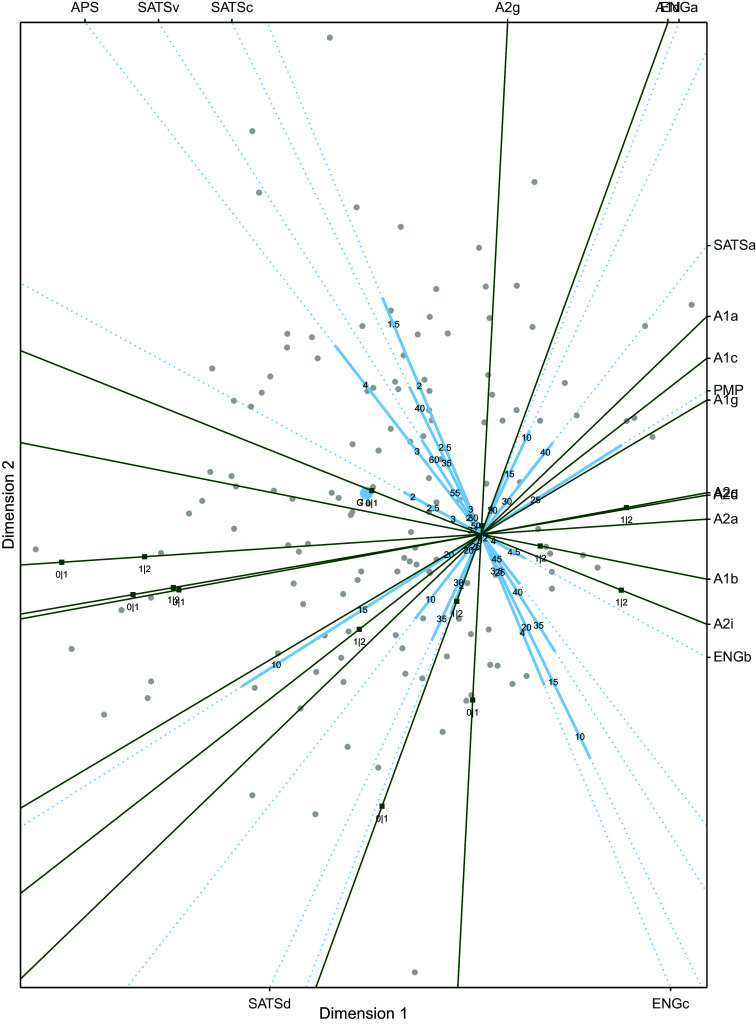
Estimated configuration for students data.*Note*: The dark green lines represent the response variables, the blue lines represent the predictor variables. Variable labels are placed on the positive side of the variables, that are the sides with the largest values. In the upper right corner the labels of ENGa and A1d overlap.

The final biplot is shown in Figure 3. The implied coefficients are shown in Table A2 (Appendix A). The figure and table can be used together to come to a interpretation of the final model. Let us first inspect the configuration of the response variables (items A1a, …, A2g) represented by the green solid lines with markers 0|1 and 1|2. By inspecting the direction of the variable axes, we see most responses are strongly correlated, that is, the variable axes have small angles and all point to the right hand side of the figure. A notable exception is item A2g, and to a smaller extent item A1d. The general pattern is that students on the right hand side of the figure correctly answer the items, while students on the left hand side often make mistakes. For items A2g and A1d, students positioned in the top of the configuration make the items correct, while at the bottom of the figure they tend to fail. Each variable axis also has two markers (0|1 and 1|2) indicating the difficulty of the item. For response variable A2a, for example, we see that these two markers are positioned strongly to the left. Projecting the students on the axes we see that most fall in the region of a correct answer. This item is relatively easy. For item A2i, however, the marker 1|2 is far to the right and the projection of student points on this axis is rarely in the region of a correct answer. Note that item A2i is also the item with the least correct answers in the data, whereas A2a has many correct responses.

Now let us further inspect the predictor variables (indicated by the light blue variable axes) and the positions of the students in the configuration. The predictor variables are represented with a variable axis that has a solid part and a dotted part: the solid part indicates the observed range in the data, from the minimum to the maximum observed value, and as such functions as a measure of effect size. The dotted part only extends the variable axis to the edge of the biplot where the variable label is placed. It can be seen that mathematical knowledge (PMP) has a long solid variable axis, that is, mathematical knowledge prior to the courses makes large differences. Students who score high on mathematics (PMP) are positioned on the right hand side. It follows that a high PMP score is predictive for answering most items correctly. Similarly, high behavioral engagement (ENGb) and a strong positive feeling about statistics (i.e., a high score on the affect scale SATSa) result in positions more to the right hand side of the biplot, where the model indicates correct responses to the items.

The predictor variable affective engagement (ENGa) points to the upper right corner, so is a good predictor for a correct response on A1d and A2g. Students who consider statistics to be a difficult topic (SATSd), in contrast, will be on the lower left corner, that is, the variable axis points in the opposite direction. Therefore, *lower scores* on this variable predict a correct response on A1d and A2g.

Higher scores on academic procrastination (APS) and the value and cognitive competence scales of attitudes toward statistics (SATSv and SATSc, respectively) result in positions more to the top left. Academic procrastination has a large effect (i.e., a long solid variable axis), whereas SATSv and SATSc have small effects (short variable axes). Points in the top left corner project low on most of the response variables, indicating wrong answers, but high (i.e., correct answers) on A2g and A1d. As such, academic procrastination and the two attitude variables have negative effects on most response items but positive effects on A2g and A1d. Finally, let us look at the gender variable. Boys represent the reference category while for girls there is a point on the left of the origin (G). When a boy and a girl have equal values for the other predictor variables, for girls we should make the jump to the left, indicating less favorable answers to the ten statistics items.

As a reviewer noted, the negative effect of the two attitude variables, SATSv and SATSc, needs more inspection as at first glance the negative effect seems a bit odd. In Fabbricatore et al. (2024), where a latent class approach was used for the statistical analysis these variables had no significant effect. We also compared our results to separately fitted proportional odds models on the ten response variables. The estimated coefficients are shown in Table A3 (Appendix A). The proportional odds model for the third response variable did not converge, therefore no coefficients are displayed. For the two attitude predictors only four coefficients have a different sign compared to the coefficients of the proportional odds model. We did not test the significance of these two predictors. Further model selection could be employed by leaving out every single variable, instead of sets of predictor variables. Alternatively, a bootstrap analysis could give further insight in the statistical significance of the retained predictors. The last rows of Tables A2 and A3 show the obtained deviances per response variable, where we see that the loss for CLRRR is negligible. We like to point out that the number of parameters estimated in the proportional odds model is 12 per response variable. For the 10 variables together, this would amount to 120 parameters, whereas our CLRRR analysis has 56 parameters, a substantial reduction.

### 4.2 ISSP data: Pro-environmental behavior

In this application and the next, we use data from the ISSP 2020, the Module on Environment (ISSP Research Group, 2022). In this first analysis, we focus on the data from Thailand ( 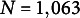 ) and the responses to four pro-environmental behavior variables, measured on ordinal scales:

1. *In the last twelve months how often, if at all, have you engaged in any leisure activities outside in nature, such as hiking, bird watching, swimming, skiing, other outdoor activities or just relaxing?* Answers on a 5-point scale: daily (coded 5), several times a week (4), several times a month (3), several times a year (2), and never (1);
2. *In a typical week, on how many days do you eat beef, lamb, or products that contain them?* Answers on a 8-point scale, 0 (coded 8), 1 (7), 2 (6), 3 (5), 4 (4), 5 (3), 6 (2), 7 (1), where numbers between brackets indicate our coding with higher numbers for more pro-environmental behavior;
3. *How often do you make a special effort to sort glass or tins or plastic or newspapers and so on for recycling?* Answers on a 4-point scale, always (4), often (3), sometimes (2), never (1);
4. *How often do you avoid buying certain products for environmental reasons?* Answers on a 4-point scale: always (coded 4), often (3), sometimes (2), and never (1).

In psychological research, the relationship between attitudes and behavior is often of interest. For these data the question is whether environmental concern (EC) and efficacy (attitudes) have an effect on behavior as measured by the four items, controlling for gender, age, and education. Furthermore, whether these effect are homogeneous for the different behaviors or heterogeneous, e.g., environmental concern may have a different effect on each of the four behavior variables. EC was measured by the following statement: *Generally speaking, how concerned are you about environmental issues*, where participants could respond on a 5-point scale ranging from “Not at all concerned” (1) till “Very concerned” (5). Environmental efficacy (EE) was measured by averaging responses to seven statements that each had a five-point answer scale ranging from Agree Strongly (5) to Disagree Strongly (1). We also use gender, age, and number of years of education as predictor variables in the analysis. We will analyze the data using both the dominance and proximity perspective and contrast the two analyses.

In Table 1, we show the fit statistics of both analyses in dimensionalities 1, 2, and 3. The AIC and BIC disagree on the optimal dimensionality, that is, AIC points to the three-dimensional solutions, while BIC points toward the two-dimensional models. We will focus on the more parsimonious two-dimensional solutions. Comparing the dominance (CLRRR) and proximity (CLRMDU) perspective, it can be seen that the fit statistics for the proximity model are a little better with lower deviance and AIC.

**Table 1:** Fit statistics for the dominance (CLRRR) and proximity (CLRMDU) analysis of pro-environmental behavior in one, two, and three dimensions

	CLRRR			CLRMDU		
Dimensionality	Deviance	AIC	BIC	Deviance	AIC	BIC
1	11,826.94	11,876.94	12,001.16	11,839.25	11,891.25	12,020.44
2	11,777.22	11,839.22	11,993.25	11,756.83	11,824.83	11,993.77
3	11,754.77	11,824.77	11,998.68	11,733.53	11,815.53	12,019.25

**Figure 4:**
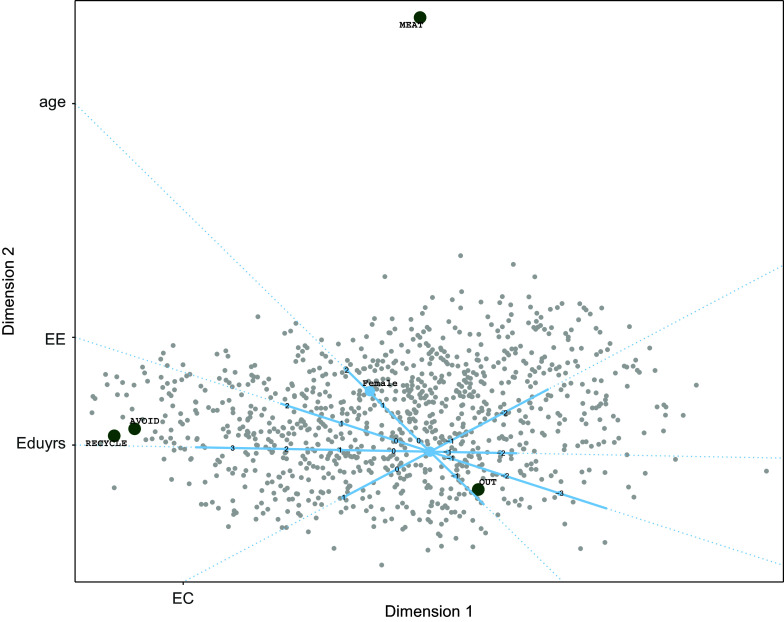
Biplot for the cumulative logistic restricted multidimensional unfolding solution relating environmental attitudes with pro-environmental behavior.

We will inspect and interpret the visualization for the proximity model, the solution of the dominance model can be found in Appendix B (we will comment on it at the end of this section). The biplot is shown in Figure 4, where it can be seen that the position of three of the four response variables is at the periphery of the configuration, while one is more centrally located (i.e., OUT). This means that, while the predictors have a dominance relationship with the response variables MEAT, RECYCLE, and AVOID, they have a proximity relationship with OUT.

**Figure B1:**
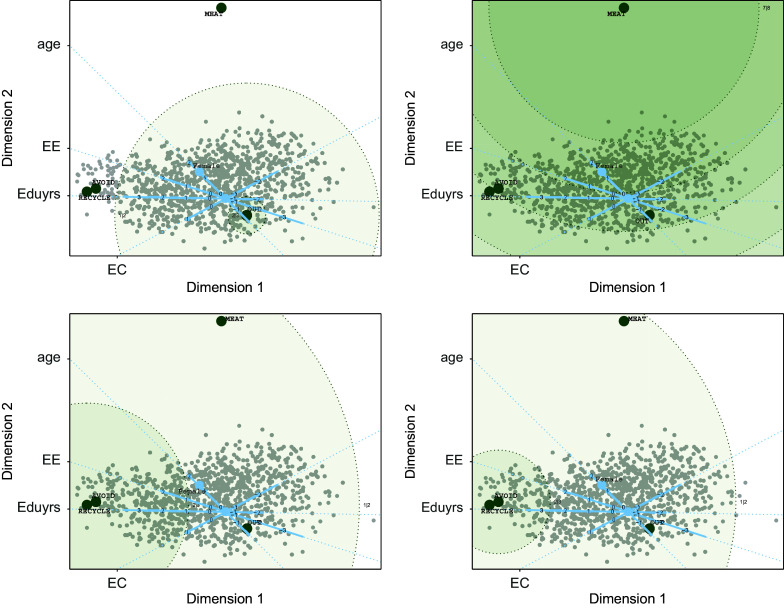
Biplot for the cumulative logistic restricted multidimensinal unfolding solution relating environmental attitudes with pro-environmental behavior.

Let us look at the biplot in more detail. The two predictor variables of interest, EC and EE, are represented by variable axes that run from the right hand side to the left hand side, meaning that persons that score high on these variables are located at the left hand side of the biplot, while persons that score low on these variables are positioned at the right hand side in the biplot. The control variables, education and age also run from right (lower scores) to left (higher scores). Females are located more to the top left compared to males. The higher a participant scores on EE or EC the closer they get to the response variables RECYCLE and AVOID, meaning that the higher a participant scores on the two attitude variables the more pro-environmental behavior is reported. For these two variables only the radii for *never* | *sometimes* (1|2) and *sometimes* | *often* (2|3) are positive, meaning that participants are never classified in the response category *always*. The biplots including the decision regions per response variable are shown in Figure B1 in Appendix B. These two response variables follow a dominance response process because all participant are located on one side of the point representing the response variable.

The response variable OUT lies close to the origin of the two-dimensional solution. The radii for boundaries *never* | *several times per year* (1|2) and *several times per year* | *several times per month* (2|3) are positive. No participant will be classified in the class of *daily* (5) or *several times per week* (4) outside activities, as the estimated radius for these boundaries are negative. Note that younger participants are closer to the point for this response variable, indicating younger people more often engage in outside activities. With increasing scores on environmental efficacy (and average values for the other variables) the behavior changes from *several times per year*, to *several times per month*, back to *several times per year*, and extrapolating to *never*, a single peaked response pattern. A similar response function can be obtained for the predictor EC.

Finally, we inspect the response variable MEAT. All radii are positive and therefore participants can be classified in each of the categories. The classification regions are shown in Figure B1, where for this data set the participants fall in 0, 1, 2, and 3 days per week eating meet (notice the reverse where not eating meat (0 days) is coded as 8 indicating the category with most pro-environmental behavior). No participant is classified as eating meat more days per week, these regions fall on the lower bottom of the figure, where no participants are located. We see that some participants will be classified as typically eating no meat (within the smallest circle around MEAT). There is not a single predictor pointing in this direction, but a combination of female, higher age, relatively low EC, and a low number of years of education will result in this classification.

**Figure B2:**
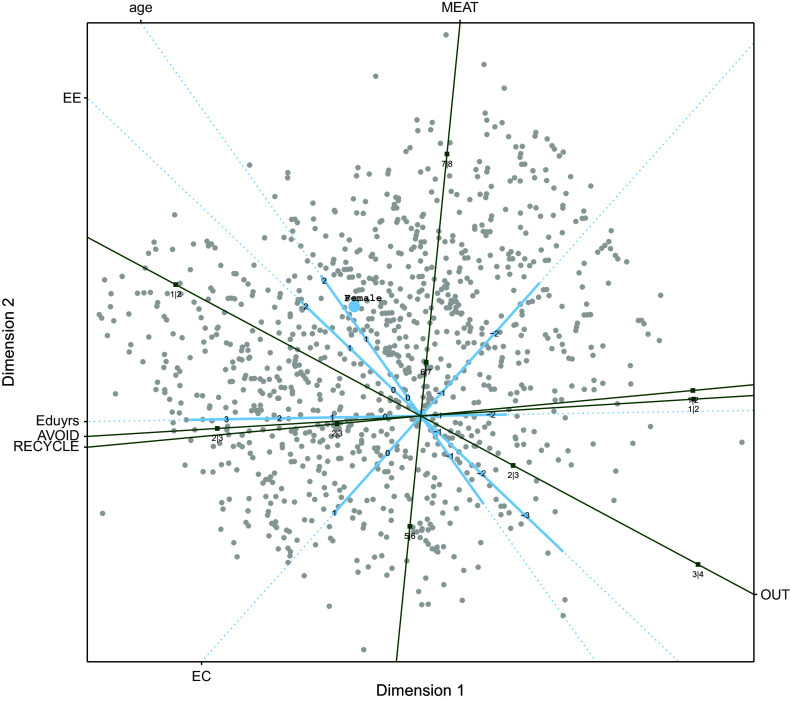
Biplot for the cumulative logistic reduced rank model relating environmental attitudes with pro-environmental behavior.

As pointed out before, the answers to three out of the four response variables follow a dominance pattern, whereas only the responses to OUT follow a proximity pattern. This response variable (OUT) is probably the reason that the proximity model fits these data better than the dominance model. The biplot for the two-dimensional dominance model is shown as Figure B2, where the interpretation for MEAT, AVOID and RECYCLE closely follows the interpretation given here. The direction of the response variables is very similar to the positions in Figure 4. The pattern for response variable OUT is different, now being monotonic and negatively related with environmental efficacy, age, and being female.

### 4.3 ISSP data: EE

In this second analysis using the ISSP data, we focus on environmental efficacy. The seven items related to environmental efficacy are:

1. It is just too difficult for someone like me to do much about the environment;
2. I do what is right for the environment, even when it costs more money or takes more time;
3. There are more important things to do in life than protect the environment;
4. There is no point in doing what I can for the environment unless others do the same;
5. Many of the claims about environmental threats are exaggerated;
6. I find it hard to know whether the way I live is helpful or harmful to the environment;
7. Environmental problems have a direct effect on my everyday life.

Participants had to indicate on a five-point Likert scale for each of these statements whether they agreed strongly (coded as 5), agreed (4), are neutral (3), disagreed (2), or disagreed strongly (coded as 1).

For these data, we like to know whether there are differences between participants from different countries concerning environmental efficacy, controlling for gender, education, and age. Therefore, as predictor variables we use the 12 zero-one coded dummy variables for the countries, using Thailand as the reference category (i.e., coded with 12 zeros), a dummy variable for gender where female is coded 1 and therefore male serves as a reference, and both the standardized scores of age and number of years of education as numeric predictors.

For the analysis of these data, we use the CLRMDU model, a proximity model that is usually considered a better choice for attitude data. With the above coding of the predictor variables, the origin of the Euclidean space corresponds to male participants from Thailand having average age and education.

We fitted models in 1, 2, and 3 dimensions. The fit statistics are

**Table:** 

Dimensionality	Deviance	npar	AIC	BIC
1	316,958.57	50	317,058.57	317,444.02
2	313,685.65	71	313,827.65	314,374.99
3	312,597.80	91	312,779.80	313,481.32

showing that the three-dimensional model fits best. For illustrative purposes, the two-dimensional biplot is shown in Figure 5. The response variables are located from the bottom left (item 1, too difficult) to top middle (item 2, do right). They lie almost on a curve, where items 5 and 6 (exaggerated and hard to know) lie close together, indicating similar response tendencies for those two items.

Looking at the predictor side we first note that the origin, where the variable axes for *Education* and *Age* cross represents male participants from Thailand with average age and education. We can see that *Education* has a large influence on the positioning of the participants, where participants with a few years of education are in the top of the biplot and those with many years of education in the bottom. The variable axis for *Age* is much shorter, and therefore *Age* has a smaller effect on the outcomes. The last control variable is *Gender*, where we see that the category Female (Fem) is below the origin. Therefore, comparing female and male participants with the same values for the other predictor variables, the females are located below male participants and therefore closer to the items at the bottom of the configuration. The countries partition in two clusters, with on the right hand side of the biplot the Asian countries Japan, the Philippines, Taiwan, and Thailand and on the left hand side a cluster of Hungary, Russia, New Zealand, Island, Slovenia, Switzerland, Austria, Germany, and Finland. Austria and Germany are very close to each other, meaning that responses in these two countries follow a similar pattern. Russia and Hungary are close together and positioned more in the top of the biplot, further away from the response variables more important, exaggerated, hard to know, and too difficult, indicating lower probabilities of agreement with these items compared to participants from other countries.

Now, let us look in more detail at two biplots where we included the circles representing the classification regions for items 4 (“There is no point in doing what I can for the environment unless others do the same”) and 7 (“Environmental problems have a direct effect on my everyday life”). The biplots are shown in Figure 6. For item 4, we see that the cluster of Asian countries on the left disagrees (all participants from Taiwan and most participants from the Philippines) or is neutral (most participants from Thailand and the higher educated and elderly in Japan).

**Figure 5:**
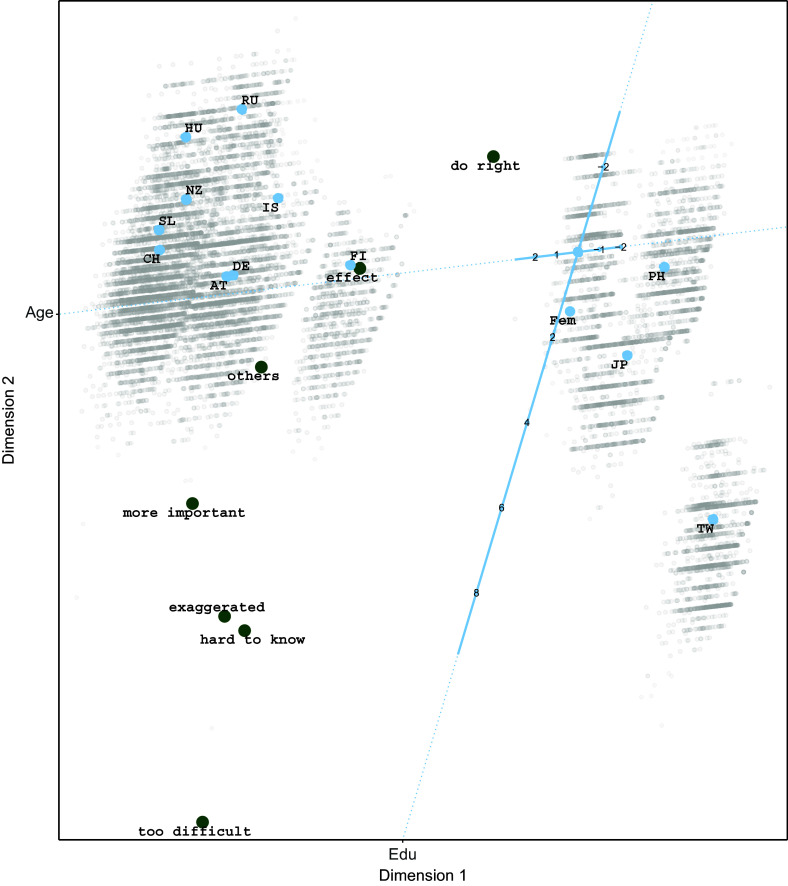
Estimated configuration for environmental efficacy data.

Participants from the other countries mainly agree with this statement, because their position is within the *neutral* | *agree* (i.e., 3|4) circle. An exception are participants from Russia and Hungary with low scores on education, who tend to be neutral.

Inspecting the biplot with classification regions for item 7, we can conclude that only participants from Finland with average age and education tend to agree with this item. Most participants from the Asian countries tend to disagree (except participants from Thailand), while most participants from the other countries tend to be neutral toward this item. It seems that especially *Age* has an influence on this response variable as elderly people from the Asian countries have larger probabilities to agree, while the younger people from the other cluster have higher probabilities to agree (i.e., they are closer to the position of the item).

## 5 Simulation studies

### 5.1 Parameter recovery

We conducted a simulation study to verify whether the algorithms work properly. Therefore, we use the estimated parameters from the second example, described in Section 4.2, as the basis for the population values. In that example, the number of response variables *R* equals 4, the number of predictor variables *P* equals 5. The population model has two dimensions ( 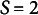 ).

**Figure 6:**
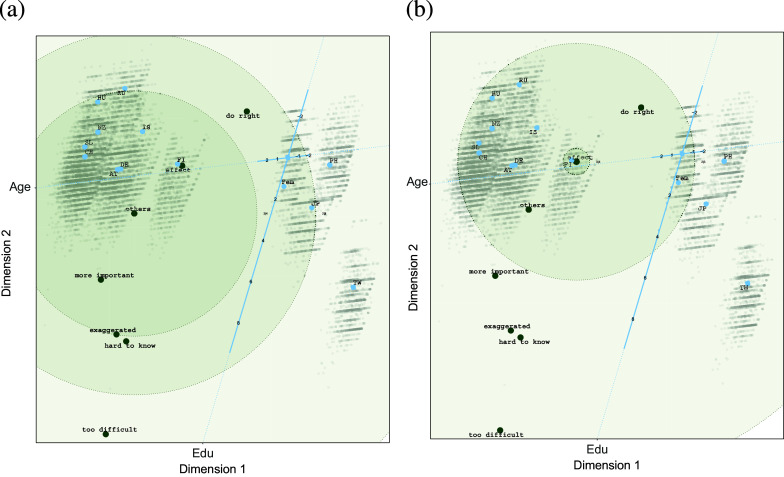
Estimated configuration for environmental efficacy data with decision regions for Item 4 (a) and Item 7 (b).

In the data generation process, we start drawing predictor variables from the multivariate normal distribution with mean zero and covariance matrix equal to the correlation matrix of the predictors in Section 4.2 (see Appendix C for values). The predictor variables and the population matrix 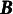 define 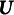 . With these coordinates and the matrix 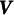 , values on the latent variables (i.e., the 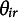 ’s) can be computed. For the dominance models we use the inner product ( 
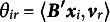
 ), while for the proximity models we use the distances ( 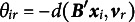 ). With these values and the threshold parameters, probabilities of the response categories for each of the response variables can be obtained. We draw observed outcome variables for each response variable independently from the multinomial distribution.

We vary sample size with values 250, 500, and 1,000. We also vary whether the response variables have three or five categories (threshold values can be found in Appendix C) and we manipulate the number of response variables, to be equal to 4 or 8. Note that in the empirical example there are four response variables. For the condition with eight response variables, we created four others by simply rotating 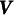 by 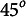 and adding the obtained coordinates to the matrix (see Appendix C for population values).

Finally, to verify whether the recovery is sensitive to the distribution of the predictor variables, we changed the distribution of the predictor variables into a uniform distribution and we categorized the predictor variables into five-point Likert scales. We used a full factorial 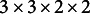 design, with 200 replications per condition. The simulation is done separately for CLRRR and CLRMDU.

As outcome variable we take the following measure of recovery: 

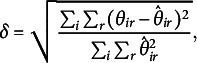

where 
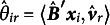
 for the dominance model and 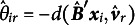 for the proximity model. This measure resembles the Stress-1 value that is often used in multidimensional scaling and unfolding. The benefit of this measure over, for example, looking at the recovery of 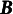 or 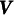 is that we do not need to take into account any reflection, rotation, or other transformations to overcome indeterminacies. We like to point out that the measure 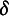 is not necessarily comparable between the dominance and proximity perspective as for the proximity models the values of 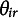 are all negative, while for the dominance models they can be both positive and negative.

**Figure 7:**
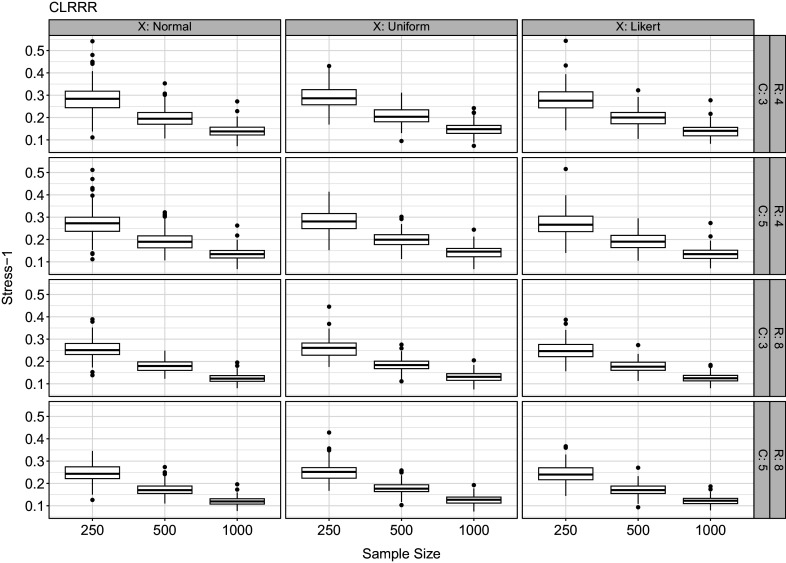
Simulation results for cumulative logistic reduced rank regression.*Note*: *R* denotes the number of response variables, *C* the number of response categories per response variable. The three columns show different distributions for the predictor variables (normal, uniform, Likert). On the horizontal axis we show the different sample sizes, while on the vertical axes, the value of recovery is found where lower values represent better recovery.

**Figure 8:**
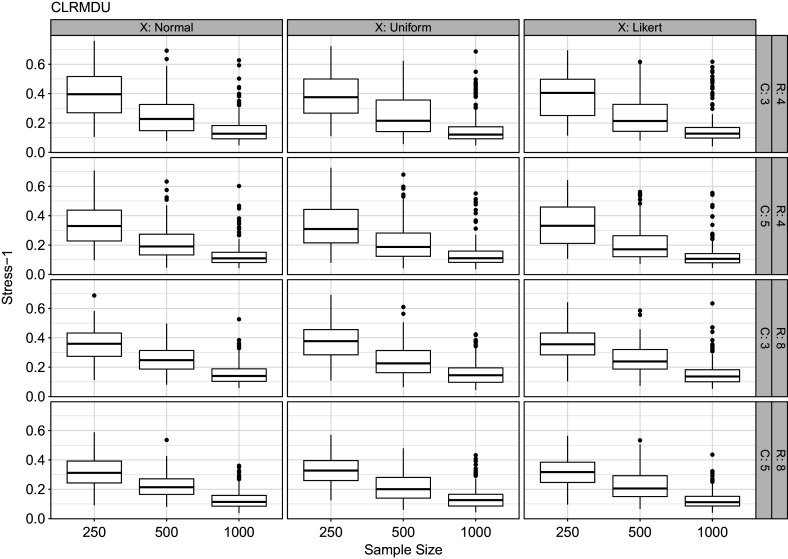
Simulation results for cumulative logistic restricted multidimensional unfolding.*Note*: *R* denotes the number of response variables, *C* the number of response categories per response variable. The three columns show different distributions for the predictor variables (normal, uniform, Likert). On the horizontal axis we show the different sample sizes, while on the vertical axes, the value of recovery is found where lower values represent better recovery.

The results of our simulation studies are presented in Figure 7 for CLRRR and Figure 8 for CLRMDU. In both cases, we see that recovery is good and that it improves with increasing sample size, number of response variables (*R*), and number of response categories (*C*). The changes for the number of response variables and the number of response categories are small, but the values of 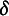 become smaller and less variable. Sample size has a larger influence as is clear from the boxplots. The distribution of the predictor variables has hardly any influence on the recovery.

Comparing the recovery of the dominance (Figure 7) with the proximity model (Figure 8), we see that the recovery for the dominance model is slightly better, that is, the values are lower and the variability is smaller. One reason might be that the proximity model sometimes ended in a local optimum as we did not use multiple starting values but simply used the population values as starting point.

### 5.2 Dimension selection

Another aspect of our modeling framework is the choice of dimensionality. In this article, we use information criteria for this choice. We conducted a small simulation study to illustrate the behavior of the AIC and BIC for CLRRR and CLRMDU.

We use the same settings as in the previous simulation study, where we only use the normally distributed predictor variables. So, we vary the number of response variables (4 or 8), the number of response classes per response variable (3 or 5), and the sample size (250, 500, or 1,000). We generated data sets with two dimensions and subsequently fitted models with one, two, and three dimensions. In each condition, we repeated this procedure 200 times and counted the number of times the information criteria select a certain dimensionality. The simulations were done separately for the two types of models. The results are shown in Table 2.

Inspecting the results for CLRRR, we see that the AIC only selects two and three-dimensional models, while the BIC only selects one or two-dimensional models. The AIC is insensitive to the sample size, that is, the number of times the three-dimensional solution is chosen does not vary much. The number of response variables or response categories does not seem to influence the selection. For BIC, the sample size matters. In small samples it tends to more often select the one-dimensional solution, especially with four response variables. In larger samples the correct dimensionality is chosen.

For CLRMDU the information criteria show different patterns. The AIC often selects the correct dimensionality. When the number of response variables and response categories are small and also the sample size is small it might select the one-dimensional solution. The BIC tends to select the one-dimensional model especially for the smaller data sets.

**Table 2:** Results of simulation studies for dimension selection

			CLRRR						CLRMDU					
			AIC			BIC			AIC			BIC		
*C*	*R*	*N*	1	2	3	1	2	3	1	2	3	1	2	3
3	4	250	0	178	22	90	110	0	83	104	13	200	0	0
3	4	500	0	170	30	5	195	0	27	150	23	198	2	0
3	4	1,000	0	173	27	0	200	0	3	179	18	172	28	0
3	8	250	0	180	20	45	155	0	32	141	27	199	1	0
3	8	500	0	168	32	0	200	0	2	183	15	175	25	0
3	8	1,000	0	174	26	0	200	0	0	188	12	22	178	0
5	4	250	0	179	21	79	121	0	47	139	14	200	0	0
5	4	500	0	175	25	3	197	0	12	167	21	188	12	0
5	4	1,000	0	168	32	0	200	0	0	182	18	91	109	0
5	8	250	0	170	30	35	165	0	6	178	16	188	12	0
5	8	500	0	175	25	0	200	0	0	185	15	111	89	0
5	8	1,000	0	173	27	0	200	0	0	189	11	3	197	0

*Note*: Numbers indicate how often a certain dimensionality is chosen by either the AIC or BIC out of 200 replications. *C* represents the number of response categories; *R* represents the number of response variables; *N* the sample size. The numbers 1, 2, and 3 beneath either AIC or BIC represent the selected dimensionalities. There are six columns for cumulative logistic reduced rank regression (CLRRR) and six for cumulative logistic restricted multidimensional unfolding (CLRMDU).

## 6 Conclusion and discussion

### 6.1 Summary of obtained results

In this manuscript, we proposed a novel framework for multidimensional analysis of ordinal data in a cumulative logistic framework. Logistic regression models are the usual models of choice when the response variable is categorical. For an ordinal response variable, the cumulative logistic regression model, also known as the proportional odds regression model, is a typical analysis model often used in practice. We developed cumulative logistic models for multiple ordinal response variables. Biplot and triplot representations of the results of these models were proposed and in the empirical examples the biplots were interpreted in detail.

We distinguished between models for proximity and dominance items. For proximity items, distance representations based on MDU are used, whereas for dominance items inner product representations based on principal components are used. The dominance models are most useful for cognitive response variables, whereas the proximity models are most useful for behavioural and attitude response variables. It is important to note that coding of the ordinal response variables matters for the proximity models. Say, we have an ordinal variable with four categories: always, often, sometimes, and never. Coding the four categories as 4, 3, 2, and 1, respectively, leads to different results than with the reverse coding 1, 2, 3, and 4. In contrast, for the dominance model reverse coding leads to the same solution, where only the sign of the coefficients change, but the fit remains the same.

When predictor variables are available for the participants, restricted versions of both types of models are obtained, leading to RRR and RMDU.

For maximum likelihood estimation, an EMM algorithm was developed and tested. In a simulation study, we tested the performance of the algorithm and we showed that overall the recovery is good. In more detail, the recovery increases with sample size, more response variables, and increasing number of response categories.

Generally, the framework gives the opportunity to analyze complex data sets having ordinal response variables with dimension reduction techniques. The response variables do not need to be indicators of underlying constructs as in psychological scales but simply may be a number of related questions on a given topic.

### 6.2 Discussion

#### 6.2.1 Model selection

When applying the proposed models to empirical data, a researcher needs to select an optimal model. Model selection in the framework consists of finding an optimal dimensionality and finding an optimal set of predictor variables. Although we estimate the models by maximum likelihood this does not ensure typical likelihood based statistics can be used as we discussed in Section 2.2. Overall, our multidimensional models can best be considered in the bias-variance trade off framework, where reducing the dimensionality increases the bias but reduces the variance and *vice versa*. The optimal model finds the sweet spot where the sum of squared bias and variance is minimized. We suggested to use information criteria for this purpose, but of course other statistics can be used, such a cross-validation or 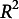 -type of statistics.

For the information criteria the number of parameters is needed for which we simply use the total number of parameters minus the number of indeterminacies (see Section 2.2). Mukherjee et al. (2015) showed, in the context of linear reduced rank models, that these numbers are naive estimates and better estimates are available. This theory, however, is not yet extended to other type of multidimensional models, such as models for ordinal response variables or distance models.

Furthermore, we suggested to first select the dimensionality and thereafter the set of predictor variables that effect the responses. Such a step-wise procedure does not guarantee that the optimal model is found. Ideally, all models with subsets of predictor variables are fitted in all dimensionalities and the best model is selected. Such an approach might become computationally very expensive. The step-wise procedure keeps the procedure simple but does not guarantee an optimal final model. More research is needed on model selection for the proposed methods.

From the visualization perspective, it is also possible to use an alternative narrative for model selection. Obviously, visualization of the results of our models is easiest when the dimensionality equals two. In that regard, one may always tend to favor the two-dimensional. The two-dimensional model is therefore the default. In our cumulative logistic multidimensional data analysis methods we simply use two dimensions unless that leads to a too large information loss. The only purpose of computing statistics like the AIC and BIC for various dimensionalities is then to verify that the information loss is not too large. What “too large” exactly means is, of course, debatable.

#### 6.2.2 Proportional odds assumption

In our multidimensional models a proportional odds assumption is made, as discussed in Section 2.1. It is not easy to test the validity of this assumption within our modeling approach. One approach would be to fit different models that do not make this assumption and compare the fit of the two models. For the CLPCA model, we could compare against multinomial multiple correspondence analysis (MMCA; Groenen & Josse, 2016). For CLRRR we could compare to a constraint version of MMCA (which first needs to be developed). Such comparisons are related to the score test for the proportional odds assumption (Agresti, 2002, Section 7.2) that tests whether the effects are the same for each cumulative logit against the alternative of separate effects. If the fit of the MMCA models is much better this is an indication of a violation of the proportional odds assumption for one or more response variables. For which response variable the assumption is violated then needs further investigation. An alternative applicable for CLRRR is to apply the score test for each response variable separately when fitting *R* different models. When none of these tests suggest a rejection of the null hypothesis, we can safely conclude that the assumption is also valid for the multivariate model. When one of the tests rejects the null hypothesis, we directly know for which response variable the proportional odds assumption is false. For the distance models (CLMDU and CLRMDU) validating the proportional odds assumption is more difficult. We could, for instance, develop new models that combine the cumulative logit models developed here with multinomial restricted unfolding (De Rooij & Busing, 2024), a distance model for nominal variables, and investigate whether such a new model signals violations of the assumption. Further work is needed to find ways to verify this assumption within the framework presented.

On the other hand, Harrell (2001) points out that we should not worry too much about the proportional odds assumption. Rank order based statistics such as the Wilcoxon test and the Kruskal–Wallis tests are special cases of the proportional odds regression model. Furthermore, rank order correlations are closely related to the proportional odds model. It seems best to place our models in a bias-variance trade-off perspective. The proportional odds assumption might not be valid for some response variables leading to biased results. However, adding parameters to avoid the assumption might lead to more variance and, when the extra variance exceeds the bias, lead to worse model performance.

#### 6.2.3 Further constraints

Sometimes, a priori information is available about which response variables group together on specific dimensions. This would entail that elements of the matrix 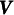 are set to zero. Similar constraints could, in theory, be imposed on the matrix 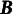 , specifying that some predictor variables are connected to specific dimensions. At the moment it is not possible to use such information in the analysis. Further research is needed to incorporate such knowledge.

In our framework, we focused on predictor variables describing the participants. In some situations, external information about the items might be available. In our analysis, we could add constraints on the matrices 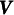 to include such information. Further research and programming is needed to incorporate such constraints.

### 6.3 Relationship to item response theory / item factor analysis

Before we conclude this article, we like to point out some relationships between our framework and item response models. Item response models have been primarily developed in the context of construction and evaluation of educational tests. To make some connections, let us start with the origins of latent variable models. For continuous response variables, there are two methods for extracting latent components from a data set: PCA and exploratory factor analysis. PCA is a general information reduction technique used in many scientific disciplines. In applied psychometrics, it is often used to extract underlying dimensions or components. PCA extracts components that explain as much variance as possible from the observed variables. In PCA, no assumption is made on the error terms, the only goal is to minimize the sum of squared errors. Factor analysis also extract components that explain as much variance of the observed variables, but the assumption is made that, given the component or latent variable, the errors are uncorrelated. This assumption is usually called the local independence assumption. As a consequence, the error terms are often called unique factors.

Both factor analysis and PCA have been generalized to the case of binary variables. Factor analysis for binary variables is often called *item factor analysis*. Takane & De Leeuw (1987) show that item factor analysis and item response models are equivalent. As discussed in the introduction, PCA has also been generalized for binary variables (De Leeuw, 2006; Landgraf & Lee, 2020; Schein et al., 2003). In these generalizations of PCA, implicitly the assumption of local independence is made, because in the log-likelihood function the contributions of the different response variables is summed. Therefore, it is not difficult to show that PCA for binary variables and item factor analysis or item response models for binary variables are equivalent as well.

Different estimation methods have been proposed for item response models (for an overview see Tuerlinckx et al., 2006), such as marginal maximum likelihood and joint maximum likelihood. In marginal maximum likelihood estimation a distribution is assumed for the person parameters (i.e., the parameters that we call 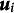 in this manuscript) and the item parameters (in our terms the 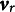 ) are estimated together with the parameters of the distribution, for example, the mean and variance of the normal distribution. In joint maximum likelihood, the person parameters are treated as fixed effects and are estimated directly together with the item parameters. In logistic PCA, the person scores are treated as fixed effects like in joint maximum likelihood. Therefore, despite the different origins, the models are very similar or equivalent.

Both item factor analysis and item response models have been developed for ordinal data. Samejima’s Graded Response Model (Samejima, 1969) is an example of a unidimensional item response model for ordinal variables. This GRM is equivalent to our CLPCA in one dimension. Multidimensional item factor or item response models (Reckase, 2009, Section 4.1.2) have been proposed in the literature that resemble our unsupervised principal component models, such as the multidimensional GRM.

In this article, we also developed supervised methods, in which predictor variables are used to explain the response variables. The cumulative logistic reduced rank model is such a model. Also in the item response model framework models have been proposed to include such person predictors (De Boeck & Wilson, 2004). In that book, Tuerlinckx & Wang (2004) proposed an explanatory variant of the unidimensional GRM for ordinal responses. Although De Boeck & Wilson (2004) lay out a general framework of explanatory item response models, advances for multidimensional ordinal data have been limited. Our supervised model for dominance items provides a multidimensional explanatory model for ordinal response variables.

Proximity item response models with a single-peaked response function have also been proposed and investigated. The best known model for ordinal data is the generalized graded unfolding model (GGUM; Roberts et al., 2000). The model definition of GGUM is quite involved, defined by two GRMs, one “from below” and one “from above.” The GGUM is a unidimensional model, no multidimensional generalizations have been proposed so far, although recently an R-package for estimation of such multidimensional models has been proposed (Tu et al., 2021). Also, recently some explanatory versions, including predictors for the observations have been proposed by Usami (2011) and Joo et al. (2022). Our (restricted) MDU models are similar to these single peaked item response models. Our model definition is, however, much simpler. More research is needed on the comparison of the two models.

For PCA, reduced rank models, and multidimensional scaling and unfolding models biplot visualizations are quite common. In contrast, biplot visualizations are quite uncommon in item response modeling. Sporadically, one sees multidimensional maps, for example, in the latent space item response model to detect item-respondents interactions (Jeon et al., 2021). The type of maps we propose in this article, might be valuable tools for (explanatory) item response modeling as well, especially for multidimensional item response modeling.

In conclusion, the framework of item response models is in some respects similar to what we proposed in this article. Although the origins of PCA and (item) factor analysis/item response theory are quite different, for categorical response variables the two approaches become similar. The focus of the two approaches, however, still differ. Item response models are usually targeted toward optimal latent trait estimation in educational or psychological measurement. Often, *a priori* knowledge is available on the traits under investigation, such as the dimensionality. External information (i.e., predictor variables) is used to address sub-population heterogeneity or to increase estimation accuracy. The goals of our analysis framework is more toward dimension reduction to obtain insight into the structure of the response variables or, when predictor variables are available, to develop simultaneous regression models for the response variables in a reduced dimensional space.

### 6.4 Software

In conclusion, in this article we proposed a family of models for multidimensional analysis of multiple ordinal response variables. The framework contains four different models. We distinguished between models for dominance variables and proximity variables. Within each we distinguished models with and without predictor variables. Algorithms for all methods proposed in this article are implemented in the R software. The logistic mapping package (De Rooij et al., 2024) contains the functions clpca and clmdu and corresponding plotting function that can be used for the analyses described in this article.

## Data Availability

The data used in this study is available from GESIS (ISSP Research Group, 2022), see https://www.gesis.org/en/issp/modules/issp-modules-by-topic/environment/2020 The data set about students performance on the statistics exam can be requested from Dr. Rosa Fabbricatore.

## Appendix A: Statistics for student data

We use different labels for the response variables than the authors in Fabbricatore et al. (2024) use. Here is a table relating our labels (left column) to the names shown in their paper (center column) and the actual question for the students:

**Table:**

A1a	T1_ClassVa_A	Select the type of variable that best describes the gross annual income
A1b	T1_GraphForQuant_A	Find the error in a graph representing the distribution of a continuous variable (i.e., waiting time in minutes)
A1c	T1_Median_A	Calculate the median of individual series data
A1d	T1_ArithmeticMean_A	Calculate the overall weighted arithmetic mean given the mean of three groups of individuals with different class sizes
A1g	T1_SkewnessNKurtosis_A	Calculate the Hotelling–Solomon skewness coefficient
A2a	T2_SamplingSpace_A	Calculate the size of the sample space if one coin and two six-sided dice are tossed together at the same time
A2c	T2_ConditionalProb_A	Calculate the conditional probability, given the marginal probabilities and the joint probability
A2d	T2_Bernoulli_A	Calculate the variance of a Bernoulli trial given the probability of success
A2g	T2_Gaussian_A	Calculate probability for normally distributed data
A2i	T2_SamplingVariance_A	Determine the sample size using the formula derived from the corrected sample variance

The model selection procedure is summarized using Table A1.

**Table A1:** Fit statistics for the student data

	npar	Deviance	AIC	BIC
1	48	2,310.47	2,406.47	2,546.98
2	74	2,250.50	2,398.50	2,615.12
3	98	2,206.37	2,402.37	2,689.24
Age	72	2,251.38	2,395.38	2,606.14
Gender	72	2,255.71	2,399.71	2,610.48
Math	72	2,277.05	2,421.05	2,631.82
Statistical.Anxiety	68	2,253.18	2,389.18	2,588.23
SATS	66	2,271.74	2,403.74	2,596.94
MSLQ	66	2,257.26	2,389.26	2,582.46
APS	72	2,258.28	2,402.28	2,613.04
AMS	72	2,250.55	2,394.55	2,605.31
ENG	68	2,265.35	2,401.35	2,600.40

*Note*: First three rows show fit statistics of models in one to three dimensions including the complete set of predictors. The remaining rows show fit statistics of two-dimensional models leaving out one variable or (set of) scales from the complete set of predictors. npar denotes the number of parameters.

The implied coefficients (  ) are shown in Table A2, and can simply be understood similarly as coefficients in a cumulative logistic model (proportional odds model).

**Table A2:** Implied coefficient from the CLRRR analysis for the student data

	A1a	A1b	A1c	A1d	A1g	A2a	A2c	A2d	A2g	A2i
0 1	0.85	2.98	4.03	1.38	2.53	2.57	0.88	2.03	1.26	0.92
1 2	1.22	0.30	0.45	0.34	1.78	2.07	0.42	1.79	0.06	1.18
G	0.03	0.61	0.19	0.00	0.17	0.68	0.30	0.61	0.27	0.96
PMP	0.05	0.37	0.29	0.38	0.23	0.55	0.27	0.54	0.45	0.47
SATSa	0.02	0.11	0.14	0.23	0.10	0.20	0.11	0.21	0.31	0.11
SATSc	0.01	0.26	0.05	0.28	0.02	0.20	0.06	0.14	0.56	0.49
SATSv	0.01	0.20	0.02	0.17	0.00	0.17	0.06	0.13	0.36	0.37
SATSd	0.02	0.06	0.13	0.23	0.09	0.14	0.08	0.16	0.34	0.02
APS	0.00	0.25	0.00	0.17	0.02	0.23	0.09	0.18	0.38	0.45
ENGa	0.01	0.02	0.07	0.13	0.05	0.07	0.04	0.08	0.20	0.00
ENGb	0.00	0.14	0.03	0.02	0.03	0.15	0.06	0.13	0.09	0.23
ENGc	0.01	0.21	0.06	0.26	0.02	0.15	0.05	0.10	0.51	0.41
deviance	290.18	234.32	206.52	253.97	154.25	162.42	297.50	165.03	245.51	251.71

*Note*: The last row shows the resulting deviance per response variable.

**Table A3:** Estimated coefficient from proportional odds models for the student data

	A1a	A1b	A1c	A1d	A1g	A2a	A2c	A2d	A2g	A2i
0 1	2.79	4.09		0.72	1.59	2.45	1.84	3.47	4.40	0.36
1 2	0.64	0.74		0.37	0.82	2.98	3.16	3.23	5.73	2.48
G	0.00	1.09		0.12	1.11	0.99	0.01	0.22	0.16	0.86
PMP	0.03	0.06		0.09	0.03	0.12	0.05	0.12	0.08	0.09
SATSa	0.03	0.01		0.01	0.05	0.00	0.02	0.06	0.05	0.01
SATSc	0.04	0.04		0.10	0.03	0.10	0.01	0.03	0.06	0.07
SATSv	0.02	0.06		0.03	0.02	0.03	0.03	0.07	0.05	0.04
SATSd	0.09	0.01		0.10	0.04	0.03	0.03	0.09	0.03	0.01
APS	0.07	0.36		0.10	0.40	0.05	0.21	0.49	0.53	0.56
ENGa	0.34	0.53		0.19	0.10	0.01	0.28	0.05	0.31	0.10
ENGb	0.12	0.03		0.73	0.70	0.86	0.09	0.09	0.00	0.19
ENGc	0.26	0.19		0.31	0.20	0.13	0.46	0.23	0.84	1.07
deviance	283.48	229.77		244.87	147.89	156.40	293.64	158.82	243.60	250.75

*Note*: The last row shows the resulting deviance per response variable.

For comparison the coefficients of 10 separate proportional odds models are shown in Table A3. The model for response variable A1c did not converge.

## Appendix B: Pro-environment behavior

The biplots for the cumulative logistic restricted multidimensional unfolding analysis including the circles for each of the response variables is shown in Figure B1.

The biplot for the cumulative logistic reduced rank model is shown in Figure B2.

## Appendix C: Population parameters for simulation studies

The predictor variables are sampled from a multivariate normal distribution with zero means and covariance matrix

**Table:**

	X1	X2	X3	X4	X5
X1	1.00	0.01	0.02	0.01	0.04
X2	0.01	1.00	0.59	0.19	0.16
X3	0.02	0.59	1.00	0.00	0.00
X4	0.01	0.19	0.00	1.00	0.25
X5	0.04	0.16	0.00	0.25	1.00

The matrix with population coefficients  equals the estimated coefficients from the example in Section 4.2, that is,

**Table:**

	1	2
X1	0.16	0.19
X2	0.37	0.04
X3	0.17	0.19
X4	0.40	0.17
X5	0.28	0.12

The population matrix  equals

**Table:**

	1	2
Y1	0.44	0.45
Y2	0.34	2.35
Y3	1.68	0.05
Y4	1.55	0.08
Y5	0.16	0.61
Y6	2.18	0.94
Y7	0.84	1.45
Y8	0.74	1.36

where we use only the first four rows when  and the complete matrix when

The parameters  are equal for the different response variables. The threshold values in case of three categories are  1.0 and  0.5, while the values with five response categories are  2.0,  1.5,  1.0, and  0.5.
